# Breaking the Silence: Exploring Self-experienced Discrimination Among Non-EU Physicians in Denmark

**DOI:** 10.1192/j.eurpsy.2025.613

**Published:** 2025-08-26

**Authors:** F. Monshizadeh Tehrani, M. Nouri, M. Hossein Pour, V. H. Santos, D. Zani

**Affiliations:** 1 Child and Adolescents Mental Health Center, Mental Health Services in the Capital Region of Denmark, Copenhagen, Denmark; 2 Zanjan University of Medical Science, Zanjan, Iran, Islamic Republic Of; 3 Pilehuset Dementia Center, Municipality of Copenhagen, Copenhagen, Denmark; 4 Department of Psychiatry and Mental Health, Cova da Beira Local Health Unit; 5 Faculty of Health Sciences, University of Beira Interior, Covilhã, Portugal; 6 Psychiatric Services Grisons, Chur; 7 Institute for Biomedical Ethics and History of Medicine, University of Zurich, Zurich, Switzerland

## Abstract

**Introduction:**

Discrimination against foreign healthcare professionals is an underexplored issue. Like many other countries, Denmark is experiencing an increasing influx of non-European Union (EU) physicians. These physicians face higher levels of requirements to obtain authorization to practice and be included in the Danish healthcare sector compared with their European peers, which can lead to an experience of perceived discrimination. Considering importance of physicians’ mental health, addressing this perceived discrimination is crucial.

**Objectives:**

This study aims to evaluate the perceived daily discrimination among non-EU physicians residing in Denmark. We focus on those who have immigrated within the past 10 years to shed light on the challenges faced during their integration into the Danish healthcare system.

**Methods:**

62 non-EU physicians who immigrated to Denmark within the last decade participated in the online survey during January 2024. The survey consisted of demographic information and Perceived Discrimination Scale (PDS). PDS was used to assess daily discrimination. Participants were grouped based on their duration of stay in Denmark and employment status. A Kruskal-Wallis H Test was conducted to compare the median daily discrimination scores across the different groups, using SPSS version 29.

**Results:**

The study revealed that 74% of the participants who had lived in Denmark for less than four years were unemployed (p-value=0.001), suggesting significant challenges in finding employment. Furthermore, participants living in Denmark for over four years reported significantly higher levels of perceived daily discrimination compared to newcomers (p-value=0.016), indicating difficulties in integration. Similarly, employed physicians, reported higher discrimination levels than their unemployed peers (p=0.053), suggesting discrimination experiences at work.

**Conclusions:**

This study reveals that non-EU physicians in Denmark face significant challenges to secure employment, especially in their first years of residence. Additionally, perceived discrimination for the physicians may be influenced by both employment status and duration of stay. High unemployment rates among recent arrivals and increased discrimination for those residing longer suggest that extended integration into the Danish healthcare system can intensify feelings of bias.

**Disclosure of Interest:**

None Declared

